# Decision upon Jury Service

**Published:** 1899-07

**Authors:** 


### DECISION UPON JURY SERVICE.

Dr. E. P. Beadles, of Danville, Va., has performed the dental profession a good service in the matter of jury duty. He was summoned, but carried the matter before another court, and secured a decision that he ranked as a physician and "was not subject to jury service." This will, doubtless, be of value to others similarly situated.

